# Biocompatibility Assessment of PLCL-Sericin Copolymer Membranes Using Wharton's Jelly Mesenchymal Stem Cells

**DOI:** 10.1155/2016/5309484

**Published:** 2015-12-29

**Authors:** Kewalin Inthanon, Donraporn Daranarong, Pimwalan Techaikool, Winita Punyodom, Vorathep Khaniyao, Audrey M. Bernstein, Weerah Wongkham

**Affiliations:** ^1^The Human and Animal Cell Technology Research Unit, Department of Biology, Faculty of Science, Chiang Mai University, Chiang Mai 50200, Thailand; ^2^Biomedical Polymers Technology Unit, Department of Chemistry, Faculty of Science, Chiang Mai University, Chiang Mai 50200, Thailand; ^3^The Graduate School, Chiang Mai University, Chiang Mai 50200, Thailand; ^4^Departments of Ophthalmology and Pharmacology and System Therapeutics, Icahn School of Medicine at Mount Sinai, One Gustave L. Levy Place, P.O. Box 1183, New York, NY 10029, USA

## Abstract

Stem cells based tissue engineering requires biocompatible materials, which allow the cells to adhere, expand, and differentiate in a large scale. An ideal biomaterial for clinical application should be free from mammalian products which cause immune reactivities and pathogen infections. We invented a novel biodegradable poly(L-lactic-co-*ε*-caprolactone)-sericin (PLCL-SC) copolymer membrane which was fabricated by electrospinning. Membranes with concentrations of 2.5 or 5% (w/v) SC exhibited qualified texture characteristics with a noncytotoxic release profile. The hydrophilic properties of the membranes were 35–40% higher than those of a standard PLCL and commercial polystyrene (PS). The improved characteristics of the membranes were due to an addition of new functional amide groups, C=O, N–H, and C–N, onto their surfaces. Degradation of the membranes was controllable, depending on the content proportion of SC. Results of thermogram indicated the superior stability and crystallinity of the membranes. These membranes enhanced human Wharton's jelly mesenchymal stem cells (hWJMSC) proliferation by increasing cyclin A and also promoted cell adhesion by upregulating focal adhesion kinase (FAK). On the membranes, hWJMSC differentiated into a neuronal lineage with the occurrence of *nestin*. These data suggest that PLCL-SC electrospun membrane represents some properties which will be useful for tissue engineering and medical applications.

## 1. Introduction

Biomaterials and stem cell-based technologies are promising, novel strategies for tissue engineering and regenerative medicine. Since clinical application requires a large number of cells, biomaterials must be designed to promote cell attachment, propagation, and directed cellular differentiation into specific lineages* ex vivo* before transplantation. A better understanding of how biomaterials control cellular mechanisms will help to improve the quality of cellular therapy. In addition, mammalian-free components continually become an important issue for cell preparation to avoid cross-species immunogenic reactivities and oncogenesis when the cells are implanted into patients. Natural proteins have been introduced into tissue engineering applications because they provide structure and properties which would be suitable for stem cell differentiation and transplantation. In this study, sericin (SC), a natural glue-like protein from* Bombyx mori* silkworm cocoons, was introduced into stem cell and biomaterial experiments. SC has been dominantly used for silk-based biomaterials studied and reported to provide an alternative source of collagen [1]. A synthetic scaffold, composed of a minimum of 90% SC, was an effective substrate for the proliferation of adherent animal cells [2] and can be used in drug delivery and the controlled release of growth factor [3]. A spongious collagen/SC scaffold enhanced adhesion and proliferation of human adipose-derived stem cells [4]. In addition, SC protein exhibited enhanced initial-attachment and proliferation of many cell types [1]. However, no studies have been published on the use of SC for human Wharton's jelly mesenchymal stem cell (hWJMSC) cultures. The study reported here is the first to examine the manipulation and cytotoxicity of SC to hWJMSC.

Human Wharton's jelly mesenchymal stem cells (hWJMSC), derived from umbilical cords, are widely used in clinical practice, regenerative medicine, and tissue engineering. They have a high proliferation rate, self-renewal capacity, and suppressed allergenic reactions and can be used without serious ethical limitations [5]. hWJMSC is a good substitute for bone marrow-derived mesenchymal stromal cells and as a source for tissue engineering and cell-based therapies [6]. They are highly pluripotent and can be differentiated into several derivatives of the three germ layers (muscle [7], bone, cartilage [5], heart [8], and brain cells [9]). However, undifferentiated hWJMSC have the greatest propensity for spontaneous differentiation into multiple lineages in standard culture systems [10] and when transplanted* in vivo* [11]. It is possible that uncommitted cells lead to abnormal differentiation and malignant formation during long-term* in vitro* culture [12], but biomaterial technologies have been introduced to overcome cell differentiation issues by controlling cell physiology including growth, differentiation, migration, gene expression, protein synthesis, and apoptosis [13].

Biomaterials provide structural stability, with or without various biochemical and biophysical cues, for developing tissues and support adhesion [13]. Some biocompatible and biodegradable scaffolds are used to replace structurally or physiologically deficient tissues and organs in humans. The most important property of scaffolds, in terms of their hierarchical structure, is the similarity of the extracellular matrix (ECM) to surrounding tissues [13]. Electrospinning has been used to fabricate biomaterials with micro- to nanoscale features [14]. Such polymeric, fibrous, meshy products have excellent flexibility with greater surface area for cell attachment. The success of fabricated materials depends on the target cells and organs [15]. Poly(L-lactic-co-*ε*-caprolactone) (PLCL) is synthetic biodegradable polyester which has been extensively used in biomedical applications [16]. This material is highly biodegradable and biocompatible for various cell types, including chondrocytes [17], osteoblasts [18], smooth muscle cells [19], and neural stem cells [20]. PLCL scaffolds also support adhesion and proliferation of the BCP-K1 (hWJMSC) cell line [21] and control cardiomyocyte differentiation of bone marrow-derived MSC in rat [22]. Many matrix types, which combine synthetic and natural materials, are able to enhance biocompatibility and biofunctionality. Their blending into synthetic polymers improves the overall cytocompatibility of the scaffolds [23]. Due to the excellent properties of both PLCL and SC, this study focuses on using SC in a cell culture system. We also reported on the design and fabrication of PLCL-SC, new biomaterials made from copolymerized electrospinning. The potential uses of the membranes for* in vitro* expansion, self-renewal, stemness maintenance, and/or differentiation of hWJMSC were also presented. The chemical profiles and biological responses of hWJMSC on PLCL-SC membranes were also determined.

## 2. Materials and Methods

### 2.1. Polymer and Sericin

PLCL 67: 33 mole% was synthesized, by Ring-Opening Bulk Polymerization (ROP) at 120°C for 72 hours, using SnOct_2_ as the catalyst [24]. Heat-degraded SC powder was purchased from the Thailand Institute of Nuclear Technology. Cocoons were cut into pieces and extracted in purified water at 120°C for 10 minutes. The aqueous solution was filtered to remove the insoluble parts and then spray-dried to form SC powder. The powder was then sterilized by gamma irradiation.

### 2.2. Fabrication of PLCL-SC Membranes

PLCL (10% (w/v)) and different concentrations of SC (0, 2.5, 5.0, 7.5, and 10.0% (w/v)) were dissolved in HFIP (1,1,1,3,3,3-hexafluoro-2-propanol (HFIP, AR grade, Sigma-Aldrich, USA)) at room temperature, using a constant, magnetic, bar stirrer (modified from Li et al. [25]). After 16–18 hours, the mixture became homogeneous and was ready to be fabricated. The PLCL-SC-blended solution was loaded into a 3 mL thermoresistant glass syringe, equipped with a 22-gauge blunted stainless-steel needle. The syringe was connected with an electrospinning apparatus and a DC power supply. The copolymer fibers were collected on an aluminum foil mat, which served as a grounded collector. The distance between the needle and the collector was fixed at 15 cm. High voltage electricity (15 kV) was applied to the needle for 30 hours when the thickness reached about 150–170 *μ*m. The electrospun PLCL-SC membranes were kept in a vacuum chamber overnight at room temperature.

### 2.3. Characteristic of PLCL-SC Membranes

#### 2.3.1. Surface Texture

The PLCL-SC membranes were characterized according to their surface texture under a scanning electron microscope (SEM; JEOL JSM-5910 LV, Oxford Instruments, UK) at an amplified voltage of 15 kV. The membranes were completely dried, using a vacuum chamber, before being mounted onto SEM stubs. The stub surface was coated with 32 to 40 nm gold particles in a sputter coat unit (SPI Module Sputter/Carbon Coater System, SPI Supplies, USA). The fiber diameter was randomly measured from SEM images with 100 fibers/image using NIH ImageJ software. The porosity of the membranes was determined over a relative pressure (*P*/*P*_0_) range of 0.18616 to 0.28685, using the Brunauer-Emmett-Teller (BET) method.

#### 2.3.2. Fourier Transform Infrared Spectrometer: FT-IR

The functional groups on PLCL-SC membranes were examined, using FT-IR (Bruker Tensor 27, USA). The absorption band was measured in transmittance mode with a spectral range of 500–4,000 cm^−1^. Approximately 1 mg of either PLCL crystal or SC powder was used as the control group. The FT-IR was performed before and after testing of membrane degradation, to verify whether functional groups still remained in the membranes.

#### 2.3.3. Differential Scanning Calorimetry (DSC)

PLCL-SC membranes were cut and weighed to obtain 3–5 mg pieces for temperature transition evaluation, including the crystallization temperature (*T*_*c*_) and melting temperature (*T*_*m*_), using differential scanning calorimetry (DSC) (DSC7, PerkinElmer Inc., USA). The thermal property measurements of PLCL-SC membranes were scanned from 0°C to 200°C, with a heating rate of 10°C per minute, under normal atmospheric conditions. Commercial-grade indium and tin were used as the reference material to calibrate the temperature.

#### 2.3.4. Swelling Property

PLCL-SC membranes, in a dried state, were precisely weighed (±0.0001 g), then immersed in a 1 mL phosphate buffer saline (PBS) solution, and incubated at ambient conditions (37°C, 5% CO_2_ and 95% relative humidity). The buffer was gently removed from the membranes, at various time intervals, in order to evaluate the weight of swollen membranes in the swelling state. Three replicates of each membrane were performed at each time interval. The percentage of swelling was calculated, using the following equation:

(1) $$\begin{array}{c} \%\text{ swelling of membrane}=\frac{W_{c}-W_{d}}{W_{d}}\times 100 \end{array}$$

(see [26]), where *W*_*c*_ is the swollen weight of the membrane after removing the buffer and *W*_*d*_ is the dried weight of the membrane before adding the buffer.

#### 2.3.5. Water Contact Angle

The hydrophilic and hydrophobic properties of the PLCL-SC electrospun membranes were investigated, using a water contact angle measurement (modified from Ji et al. [27]). The membranes were cut into pieces of 0.5 cm^2^ and attached to the cleaned glass slide. 10 *μ*L of deionized water was dropped onto the surface of each sample with a micropipette technique. Images were captured and the water contact angles (in degrees °) were analyzed by using a Holmarc's contact angle meter (HO-IAD-CAM-01, India). Measurements were performed at 25°C and 65% relative humidity. Mean values of five readings were calculated for each sample (*n* = 5).

### 2.4. *In Vitro* Hydrolytic Degradation, SC Releasing, and pH Changing Profiles of PLCL-SC Membranes

The membranes were immersed in PBS at time intervals of 1, 3, 5, 7, 9, 11, 13, 15, 20, and 30 days and incubated under ambient conditions. Any remaining PBS residues were eliminated, by rinsing the membranes with deionized water, followed by drying in a vacuum. The dried membranes from each time interval were weighed and the percentage of weight loss (% weight loss) was calculated by using the following equation:

(2) $$\begin{array}{c} \%\text{ weight loss}=\left[\;\left(\;\frac{W_{0}-W_{t}}{W_{0}}\;\right)\;\right]\times 100 \end{array}$$

(see [28]), where *W*_0_ and *W*_*t*_ are the weight of membranes before and after being immersed in the PBS, respectively, at each time interval.

The PBS from each sample was used for evaluating SC release, by measuring absorption at 280 nm, using a UV/VIS spectrophotometer (Lamda25, PerkinElmer Inc., USA). Then, the pH value of the PBS solution was measured, using a pH meter (Radiometer, Copenhagen, Denmark), according to the manufacturer's instructions. PLCL-SC membrane profiles were obtained from three replicates of each sample at each time interval.

### 2.5. Culture of Wharton's Jelly Mesenchymal Stem Cells (WJMSC): BCP-K1

BCP-K1 (hWJMSC line) was obtained from the Human and Animal Cell Technology Research Laboratory, Department of Biology, Faculty of Science, Chiang Mai University, Thailand. The cells were cultured in a complete medium, supplemented with 10% (w/v) fetal bovine serum and essential growth factors and maintained as described in Tunma et al. [29]. Briefly, the medium was replaced every 2 days; trypsinization was performed when cell density reached around 70–80% of the culture surface. All tissue culture consumables were purchased from Thermo Scientific (USA), unless otherwise stated.

### 2.6. Cytotoxicity of SC

A 1% (w/v) SC stock solution was prepared by completely dissolving SC powder in deionized water at 85°C for 30 minutes. The solution was filtered through a 0.2 *μ*m filter (Minisart Sartorius, Göttingen, Germany). BCP-K1 was plated at 3,000 cells per well onto 96-well plates and incubated under ambient conditions. After 24 hours, the medium was changed and the cells were exposed to various concentrations of SC in the medium for either 24 or 120 hours. Cell viability was evaluated with Vybrant MTT Cell Proliferation Assay Kit (Molecular Probes, Leiden, Netherlands), using a microplate spectrophotometer at OD_570_ nm. Five independent replicate experiments were performed.

### 2.7. *In Vitro* Biocompatibility of PLCL-SC Membranes

#### 2.7.1. PLCL-SC Membranes Preparation

Two types of membrane were chosen: PLCL-2.5SC (2.5% (w/v) SC) and PLCL-5SC (5% (w/v) SC) blended membrane. These membranes were cut into circular shapes of 55 mm diameter and then placed into each well of the 96-well culture plate. Sterilization was made by UV light for 15 minutes, prior to fitting onto the bottom of the other plates for cell seeding.

#### 2.7.2. SC Release from PLCL-SC Membranes

The sterilized PLCL-SC membranes were placed onto the bottom surface of the 96-well culture plates, submerged in 100 *μ*L PBS, and incubated under the ambient conditions for 24, 72, and 120 hours. The amounts of SC protein dissolved in the solution were evaluated by using a Quick Start Bradford protein assay kit (Bio-Rad Laboratories, Inc., CA, USA) with optical density at 595 nm. Bovine serum albumin (BSA) (Bio-Rad Laboratories, Inc., CA, USA) was used as a control to make a protein standard curve. The concentration of SC was calculated, relative to the formula from the standard curve. Five replications were performed on each sample.

#### 2.7.3. Cell Adhesion and Proliferation Assay

BCP-K1 (at density 30,000 cell/mL) was seeded onto PLCL-SC membranes in the complete medium, supplemented with 100x antibiotics-antimycotics solution (Sigma-Aldrich, USA). Cell adhesion and proliferation were determined after culture initiation for 24, 72, and 120 hours by using the MTT assay as described previously. Expression of adhesion protein, focal adhesion kinase (FAK), and proliferation protein, cyclin A, was evaluated by an enzyme-linked immunosorbent assay (ELISA). Briefly, the cells were fixed in 4% (v/v) formaldehyde and incubated for 30 minutes at room temperature. The membranes were washed 3 times in ice cold washing buffer or PBS-T (0.05% (v/v) Tween 20 (Sigma-Aldrich, USA) in 1x PBS). Each washing step was performed for 5 minutes, with gentle rocking. The samples were incubated with the quenching buffer (1x PBS containing 1% (v/v) H_2_O_2_ (Sigma-Aldrich, USA) and 0.1% (v/v) sodium azide (NaN_3_) (Sigma-Aldrich, USA)) for 20 minutes at room temperature. The quenching buffer was discarded, before washing the samples twice with PBS-T. The prewarmed antibody blocking buffer (5% (v/v) BSA (bovine serum albumin) (Invitrogen, USA)) was added and incubated at room temperature for 1 hour and then washed twice with PBS-T. After that, the diluted anti-FAK primary antibodies (1 : 500, mouse monoclonal antibody) or primary anti-cyclin A antibody (1 : 500, mouse monoclonal antibody) (Sigma-Aldrich, USA) was soaked into the membranes and incubated at 4°C overnight before washing with PBS-T for 3 times. The HRP (horseradish peroxidase) labeled secondary antibody (1 : 2,000) was added in each sample, incubated for 1 hour at room temperature. The alkaline phosphatase substrate for HRP,* o*-phenylenediamine (OPD tablet, Invitrogen, USA), was freshly prepared by dissolving in 12 mL of citrate-phosphate buffer, pH 5.0, containing 0.03% (v/v) hydrogen peroxide (H_2_O_2_) and incubated with the samples for 15 minutes. Then the stop reagent (3 M H_2_SO_4_) was added prior to the measurement at OD_492_ nm using a microplate spectrophotometer. Five replications were performed for each sample condition.

#### 2.7.4. Cell Morphology under Scanning Electron Microscope (SEM)

BCP-K1 was cultured on PLCL-2.5SC and PLCL-5SC membranes for 0.5, 1, 2, 4, 8, 12, 24, 72, and 120 hours, before removing the complete medium. The samples were gently rinsed twice with PBS and then fixed with 2.5% (w/v) glutaraldehyde (Sigma-Aldrich, USA) for 30 minutes at room temperature. The samples were dehydrated through a graded series of ethanol solutions (25, 50, 70, 90, 95, and 100% (v/v)) for 5 minutes at each concentration and then completely air-dried at room temperature. The dried samples were mounted on copper stubs and coated with gold in a sputter coater. The samples were then examined, using the SEM as described above.

#### 2.7.5. Characterization of Pluripotency and Differentiation

BCP-K1 was cultured on PS, PLCL-2.5SC, and PLCL-5SC membranes for 24 and 120 hours before RNA extraction (NucleoSpin RNA II, Fisher Scientific, Ireland). Total mRNA was reverse-transcribed to cDNA, using a Phusion RT-PCR kit (Thermo Scientific, USA). Then, 500 ng of cDNA was used as template, to amplify different genes using the primers listed in Table 1. Two gene groups were evaluated in this study: (1) pluripotency-related genes,* POU5F1* and* SSEA-4*, and (2) differentiation-related genes,* nestin*,* COL2A1*, and* PPAR*_*γ*_ *2*. Semiquantitative PCR was performed by using Phusion High-Fidelity PCR Kit (Thermo Scientific, USA) with subsequent thermocycler setting: denaturation at 95°C, annealing from 53 to 65°C (depending on the melting temperature of the primers), and extension at 72°C. The PCR products were detected using 1% (w/v) agarose gel electrophoresis and imaged under the UV-transilluminator. Band intensity was analyzed, using Quantity One V.4.4.1 (Bio-Rad Laboratories, Hercules, CA, USA). Expression of each gene was evaluated and normalized with the housekeeping gene,* GAPDH*. For each cell culture condition, three independent samples were analyzed.

### 2.8. Statistical Analysis

Student's *t*-test followed by multiple comparison analysis was performed to determine significant differences (*p* ≤ 0.05) among the mean values of the samples. All data was presented as mean ± standard deviation (SD).

## 3. Results

### 3.1. Characteristic of PLCL-SC Membranes

#### 3.1.1. Surface Texture

**Figure 1:**
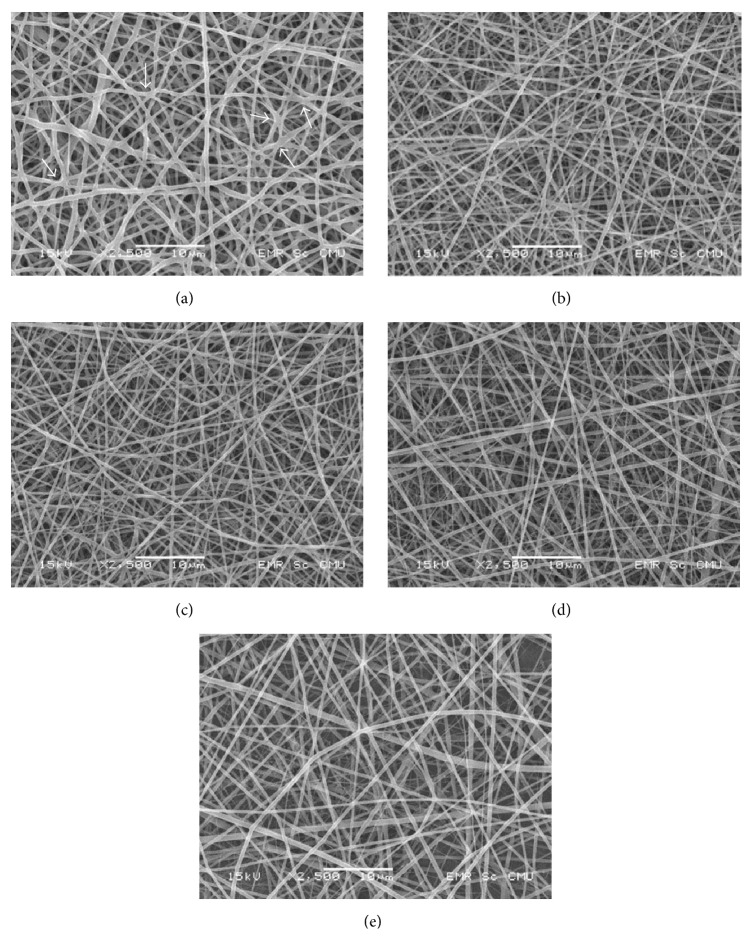
Photomicrographs of (a) PLCL 67: 33% mol solution, (b) PLCL-2.5SC, (c) PLCL-5SC, (d) PLCL-7.5SC, and (e) PLCL-10SC electrospun blended membranes (scale bar 10 *μ*M). Interfiber contacted merging (IFCM) is indicated on (a) by the arrow heads.

The PLCL-SC was homogenously blended by using HFIP, similar to that previously described by Li et al. [25]. SEM photomicrographs of all membranes revealed smooth fibers and beadless, porous, and nonwoven patterns, due to the random distribution of the fibers (Figure 1). The membrane surface was rough and white. Among the 5 types of membranes, fiber diameters of PLCL were the largest and most constant with interfiber contact merging (IFCM) (Figure 1(a)). PLCL-2.5SC (Figure 1(b)) displayed similar surface characteristics to those of PLCL, but with smaller and finer fibers, less IFCM, and higher fiber density. The size, fineness, IFCM, and density of the fibers were directly related to the amount of SC in the blend (Figures 1(c)–1(e)). Less IFCM was found with higher concentrations of SC. Fiber diameter and the membrane porosity clearly depended on the concentration of SC (Table 2). Consequently, PLCL-2.5SC and PLCL-5SC were chosen for further investigations, due to their smaller fiber diameter, higher fiber density, and their noncytotoxic concentrations of SC (see cytotoxic experiment).

#### 3.1.2. Fourier Transform Infrared Spectrometer: FT-IR

**Figure 2:**
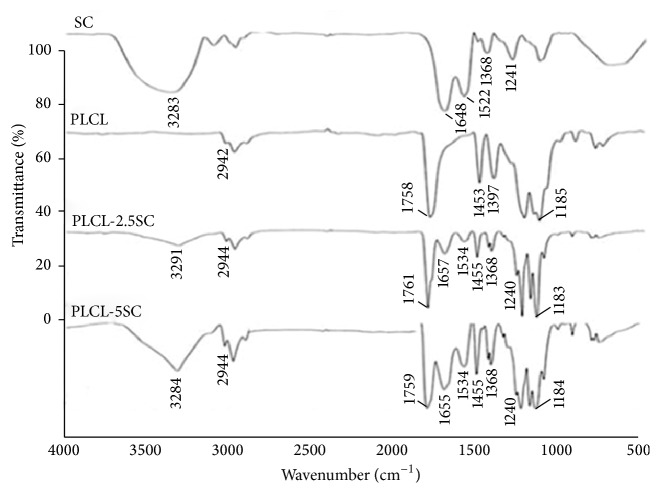
FT-IR spectra of (a) SC powder, (b) PLCL membrane, (c) PLCL-2.5SC, and (d) PLCL-5SC membranes. The peaks were analyzed to the functional groups as shown in Table 3.

PLCL-SC electrospun membranes were analyzed using FT-IR in order to verify the incorporation of SC into PLCL fibers. The spectrums from SC, PLCL, PLCL-2.5SC, and PLCL-5SC membranes exhibited peaks (cm^−1^) as shown in Figure 2. The peaks were analyzed according to the database of Kong and Yu [30] and presented specific functional groups as shown in Table 3. The PLCL-2.5SC and PLCL-5SC membranes showed the major peaks obtained from SC at 3282, 1648, 1522, and 1241 cm^−1^ which indicated the functional groups of N–H stretching, amide I, amide II, and amide III and also the peaks from PLCL at 2942, 1758, 1453, and 1185 cm^−1^ representing the groups of C–H stretching, C=O stretching, CH_2_ bending, and C–O stretching, respectively. Results indicated that the membranes contained both SC and PLCL components.

#### 3.1.3. Differential Scanning Calorimetry (DSC)

The thermal analysis revealed the influence of SC on *T*_*m*_ of the PLCL copolymer (Table 4). *T*_*m*_ and *T*_*c*_ of PLCL-2.5SC and PLCL-5SC were slightly higher than those of PLCL. Also the *T*_*m*_ of PLCL-5SC was higher, but *T*_*c*_ was lower than those of PLCL-2.5SC. Alteration of the temperature may have influenced the hydrophilic properties and directly affected the membranes' swelling properties and water contact angle (described below).

#### 3.1.4. Swelling Property

**Figure 3:**
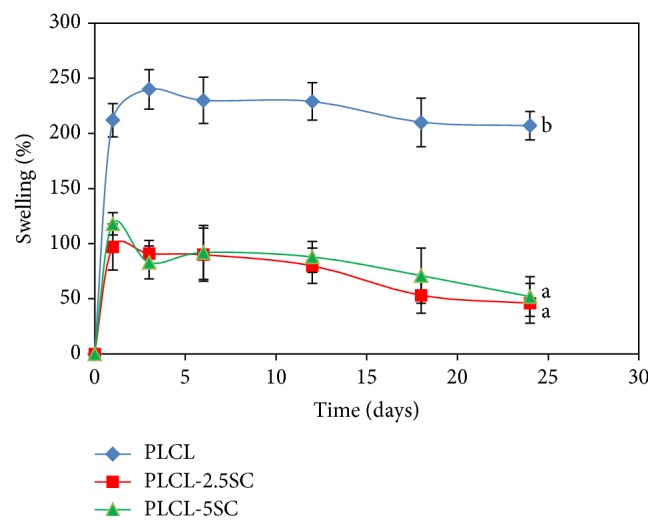
Swelling percentages of the PLCL-SC membranes over time. Statistical difference is indicated by lowercase letters (a, b) (*p* ≤ 0.05).

Swelling percentages of PLCL, PLCL-2,5SC, and PLCL-5SC either were almost stable or slightly decreased until the end of the experiment (24 days) (Figure 3). Even though highest swelling percentages of both PLCL-SC membranes were observed on day 1, they were still significantly lower than those of PLCL.

#### 3.1.5. Water Contact Angle

**Figure 4:**
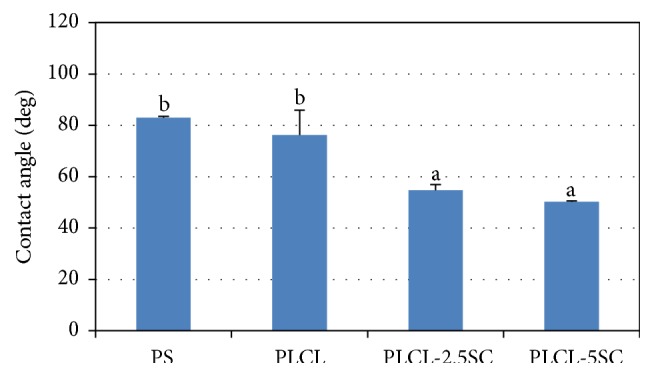
Water contact angle of the membrane surfaces. Statistical difference is indicated by lowercase letters (a, b) (*p* ≤ 0.05).

The water contact angle measurements confirmed the hydrophilic or hydrophobic degree of the membranes; the higher the degree of contact angle, the greater the hydrophobicity. The degree of angle obviously declined with higher SC concentration, resulting in significantly increased hydrophilicity (Figure 4).

### 3.2. *In Vitro* Hydrolytic Degradation, SC Releasing, and pH Changing Profiles of PLCL-SC Membranes

**Figure 5:**
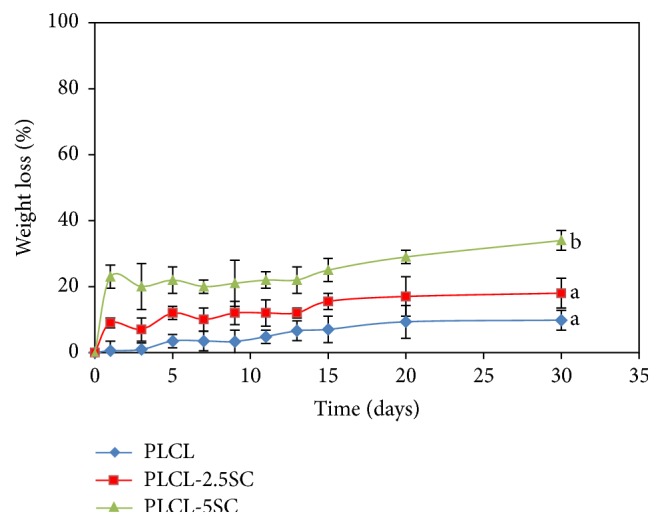
Percent of weight loss of membranes, submerged in PBS, over 30 days at 37 ± 1.0°C. Statistical differences between each group are represented as the lowercase letters (a, b) (*p* ≤ 0.05).

The degradation of PLCL-SC copolymers was assumed to be a result of random chain scission. Degradation increased with increasing SC (Figure 5). Apparently PLCL-SC membranes rapidly degraded leading to the increase of percentage weight loss in the initial stage of dipping into PBS from day 1. Thereafter, the hydrolytic degradation of the membranes changed only slightly, which was observed until the end of the experiment.

**Figure 6:**
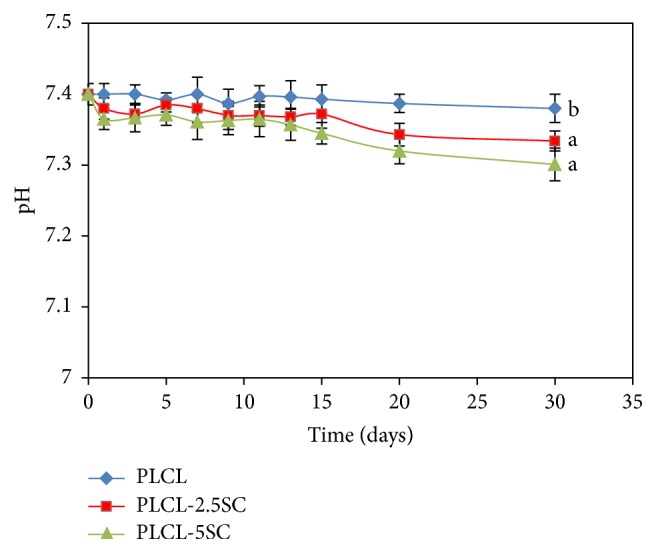
pH changing profile of the membranes submerged in PBS for 30 days at 37 ± 1.0°C. Statistical differences between each group are represented as the lowercase letters (a, b) (*p* ≤ 0.05).

The pH of PBS containing submerged PLCL membranes remained mostly unchanged or slightly declined over 30 days (Figure 6).

Membranes immersed in PBS underwent various physical changes from day 15. The color of the buffer solutions in which PLCL-2.5SC and PLCL-5SC were immersed turned to a pale-clear yellow (data not shown), since SC dissolved out of the membranes into the buffer solution. After day 15, the buffer progressively turned to be more turbid yellow, with the occurrence of sparsely suspended sediment up to day 30. No color change or sedimentation occurred with the PLCL immersed in PBS.

**Figure 7:**
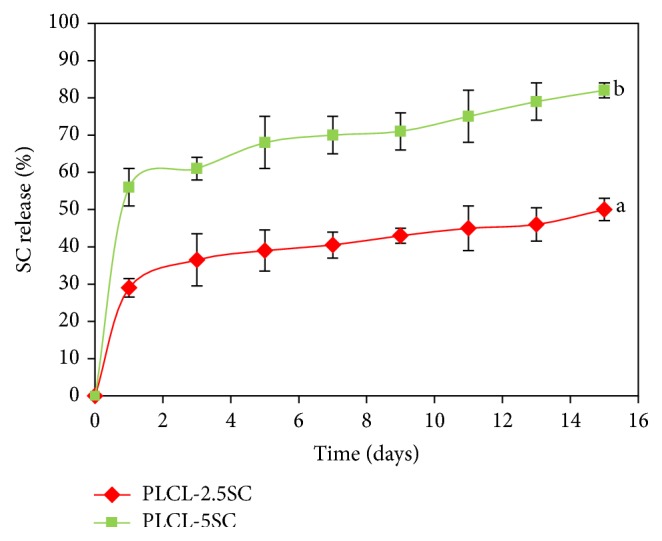
Release profile of SC from PLCL-SC membranes. Statistical differences between each group are represented as the lowercase letters (a, b) (*p* ≤ 0.05).

The highest release rate of SC was observed on the first day (Figure 7), but with the rate gradually becoming slower thereafter. It was approximately twice as high with PLCL-5SC, compared with PLCL-2.5SC, since the concentration of SC was twice as high as that in the former.

### 3.3. Cytotoxicity of SC

**Figure 8:**
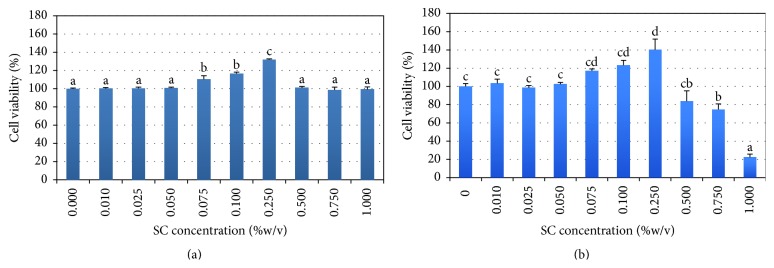
Percentages of cell viability at (a) 24 and (b) 120 hours. Statistical difference is indicated by the lowercase letters (a, b, c, and d) (*p* ≤ 0.05).

SC concentration ranging from 0.075% to 0.25% (w/v) increased the number of viable cells by 24 and 120 hours (Figures 8(a) and 8(b)). Concentrations below 0.05% promoted neither cell viability nor cytotoxicity. SC at concentrations more than 0.25% (w/v) was very harmful and toxic to the cells in a dose- and time-dependent manner. Therefore, SC must be applied to the cell culture systems as a proliferation enhancer, in accordance with the appropriate concentration and incubation time, that is, 0.075–0.25% w/v and <120 hours, respectively.

### 3.4. *In Vitro* Biocompatibility of PLCL-SC Membranes

#### 3.4.1. SC Release from PLCL-SC Membranes

**Figure 9:**
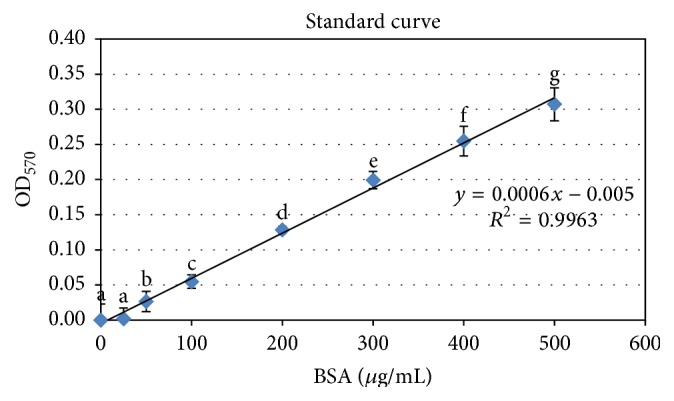
Standard curve was obtained by using the Bradford Protein assay procedure. Statistical differences between each group are represented as the lowercase letters (a–g) (*p* ≤ 0.05).

**Figure 10:**
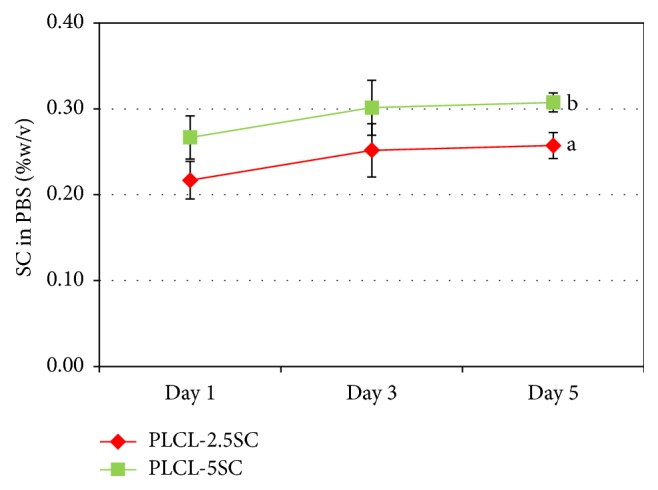
Concentrations of SC (%w/v) from PLCL-2.5SC and PLCL-5SC on days 1, 3, and 5 derived from the standard curve in Figure 9. Statistical differences between each group are represented as the lowercase letters (a, b) (*p* ≤ 0.05).

The amount of SC released from the membranes into the medium was evaluated by BSA assay. The concentration of SC dissolved in PBS was calculated by using the equation derived from the BSA standard curve (Figure 9). The dissolved concentration of SC from the PLCL-2.5SC and PLCL-5SC membranes ranged from 0.21 to 0.26 and from 0.27 to 0.31% (w/v), respectively (Figure 10). These concentrations were obviously with the ranges that enhance cell proliferation on day 1 and day 5 (Figures 8(a) and 8(b)). Consequently, PLCL-2.5SC and PLCL-5SC were used in comparison as matrices for experiments on cells.

#### 3.4.2. Cell Adhesion and Proliferation

**Figure 11:**
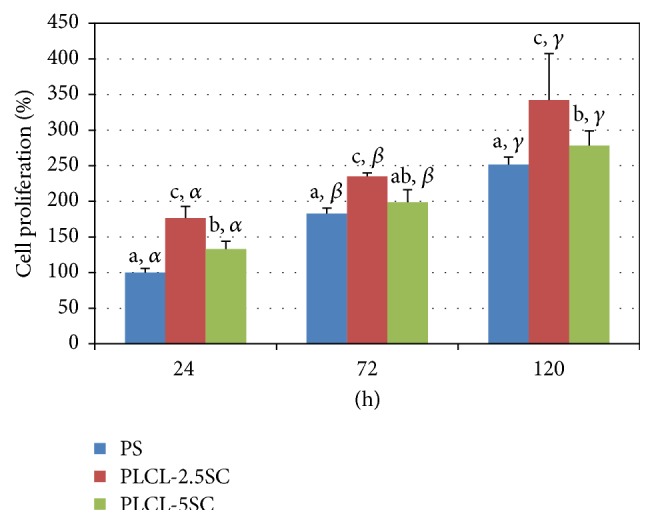
Percentages of cell proliferation of BCP-K1 on PLCL-2.5SC and PLCL-5SC membranes, evaluated by calculating the OD_570_ values from MTT assay, compared to the PS surface at 24 hours. All data presented are in mean ± SD format. Statistical difference is indicated by lowercase letters (a, b, c, and d) for each membrane on the same day. The symbols *α*, *β*, and *γ* indicate the difference between each day of the same membrane (*p* ≤ 0.05).

**Figure 12:**
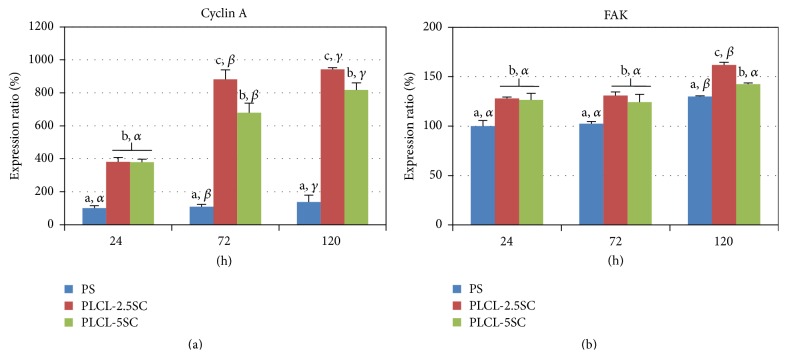
Percentages of (a) cyclin A and (b) FAK relative expression of BCP-K1 on PLCL-2.5SC and PLCL-5SC membranes examined by ELISA compared to the PS surface at 24 hours. All data are mean ± SD. Statistical difference is indicated by lowercase letters (a, b, and c) for each membrane on the same day. The symbols *α*, *β*, and *γ* indicated the difference between each day of the same membrane (*p* ≤ 0.05).

Cell attachment and proliferation enhancement on PLCL-SC membranes were verified by the upregulation of protein markers, FAK and cyclin A, respectively. FAK is involved in cellular migration, attachment, and differentiation, whilst cyclin A is a multiple cell-cycle, regulator protein, which interacts with cyclin-dependent kinase (cdk-1) and is expressed during cell division [31]. Cells preferred to adhere and proliferate more on PLCL-2.5SC than on PLCL-5SC and PS, respectively (Figure 11). These positive responses corresponded to the expression of cyclin A, which was highly upregulated on the cells from PLCL-2.5SC and PLCL-5SC on every day of culturing (Figure 12(a)). The level of cyclin A was highest in the culture of PLCL-2.5SC. This may have been because of the effective and sufficient concentration of SC released from the PLCL-2.5SC to enhance cell proliferation as described above. PLCL-5SC membranes released SC at slightly higher concentrations than PLCL-2.5SC and slightly higher than 0.25% (w/v), the precritical cytotoxic concentration (Figures 8(a) and 8(b)). In addition, the attachment protein, FAK, was highly expressed on both PLCL-2.5SC and PLCL-5SC at 24 and 72 hours of culturing (Figure 12(b)). However, the better membrane for cell attachment was PLCL-2.5SC with the highest FAK expression at 120 hours of culturing.

#### 3.4.3. Cell Morphology under Scanning Electron Microscope (SEM)

**Figure 13:**
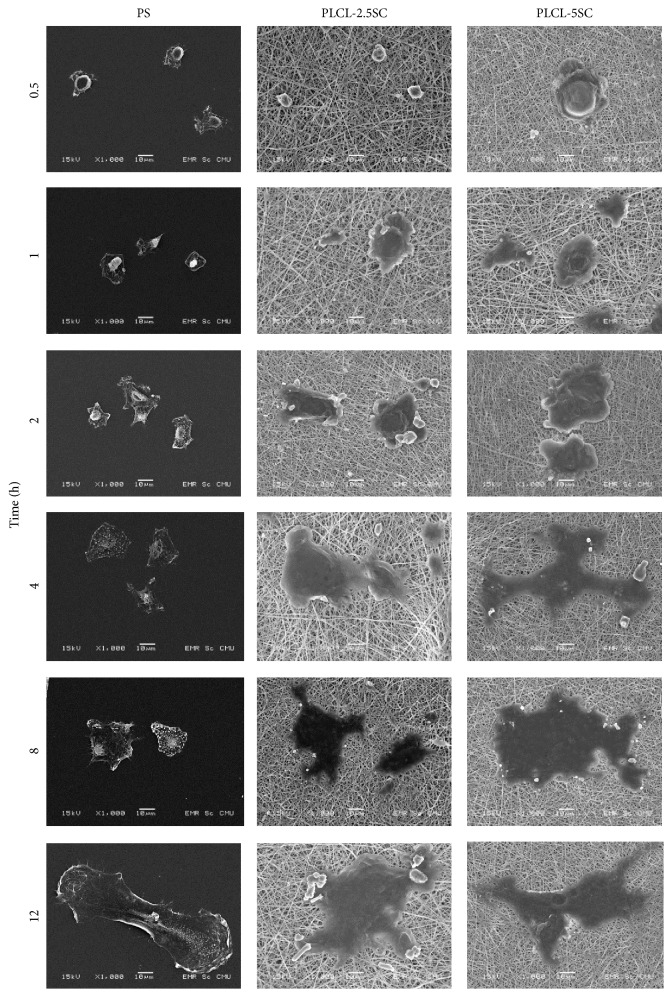
Photomicrographs from SEM showing morphology and attachment patterns of BCP-K1 on PS, PLCL-2.5SC, and PLCL-5SC membranes, cultured at 0.5, 1, 2, 4, 8, and 12 hours. The power of magnification is 1000x at 15 kV (scale bar 10 *μ*m).

**Figure 14:**
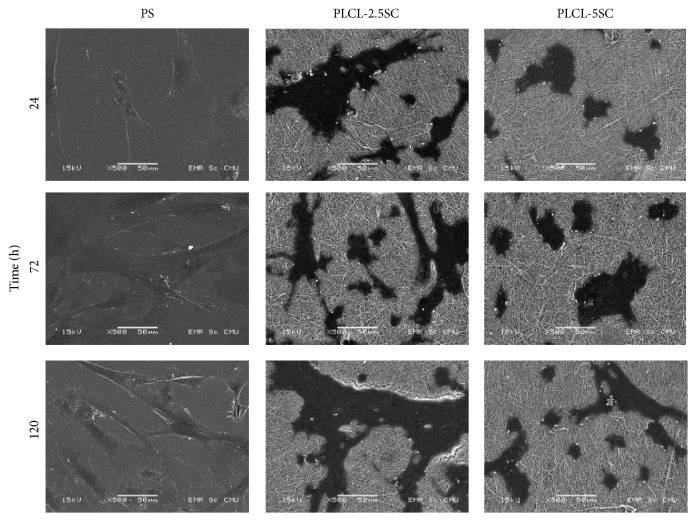
Photomicrographs from SEM showing the morphology and proliferation pattern of BCP-K1 on PS, PLCL-2.5SC, and PLCL-5SC membranes, cultured at 24, 72, and 120 hours. The power of magnification is 500x at 15 kV (scale bar 50 *μ*m).

The patterns of cellular morphology and attachment on each surface are shown in Figures 13 and 14. On the PS, cells started to attach after 0.5 hours. Cytoplasmic expansion was observed thereafter (Figure 13). From 24 hours, cells exhibited the normal mesenchymal phenotype: a flat, 2D, spindle, and multigonal shape (Figure 14). Attached cells continually proliferated at 72 and 120 hours. Cells attached to PLCL-2.5SC were round with no cytoplasmic expansion at 0.5 hours (Figure 13). Irregular-cytoplasmic spreading was observed about an hour after seeding. In contrast, cells attached to PLCL-5SC and started enlarging their cytoplasm 0.5 hours after seeding. Attached cells on both types of membrane were noticeably larger than on the PS. Multilayer piles (or colonies) of proliferated cells were observed from 8 to 120 hours. The irregular cell morphology may have been according to the degraded surface and erosion of the membranes, due to dissolving of SC. Initially attached cells subsequently migrated deeper into the membrane and once cells started dividing, they subsequently grew on top of each other.

#### 3.4.4. Characterization of Pluripotency and Differentiation

**Figure 15:**
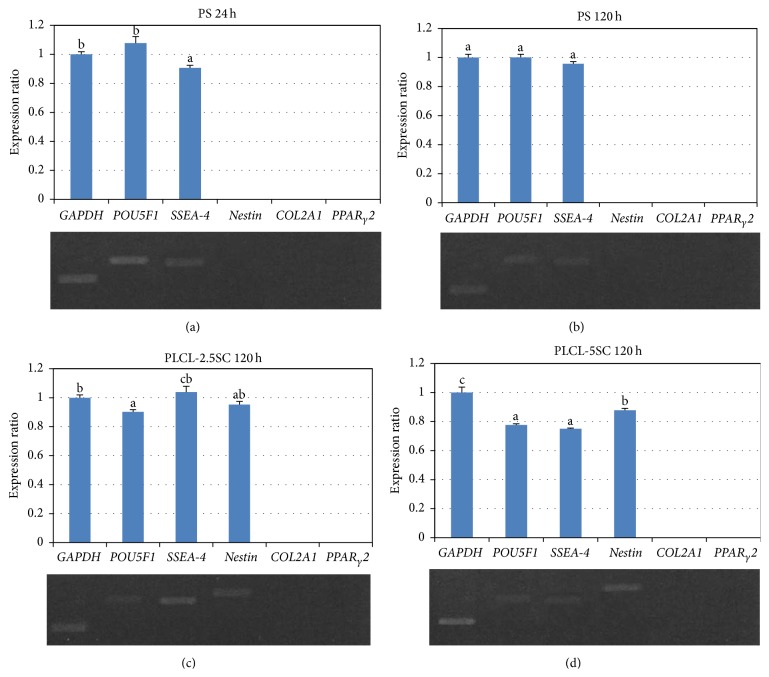
Gene expression of BCP-K1 cultured on PS (a) 24 hours, (b) PS 120 hours, (c) PLCL-2.5SC 120 hours, and (d) PLCL-5SC 120 hours. Statistical difference is indicated by lowercase letters (a, b, and c) (*p* ≤ 0.05).

The maintenance of pluripotency was evaluated by the expression of the embryonic stem cell markers*, POU5F1* and* SSEA-4* [32, 33]. In parallel, differentiation-related genes,* nestin*,* COL2A1*, and* PPAR*_*γ*_ *2*, the differentiation markers for neuronal, chondrogenic, and adipogenic differentiation, respectively [34–36], were also assessed. The experimental results revealed that cells on PS expressed a high level of* POU5F1* but a low level of* SSEA-4* over 24 hours (Figure 15(a)). The expression patterns showed no significant change up to 120 hours (day 5) (Figure 15(b)). There was no detectable PCR production of* nestin* at either 24 or 120 hours on PS. The cells could possibly maintain their pluripotent stages in culture, over the time periods used in the experiments. On PLCL-2.5SC, cells expressed both pluripotency and differentiation-related genes, with a higher expression level of* SSEA-4* than* POU5F1* and* nestin* (Figure 15(c)). The expression of* nestin* was much higher than the pluripotency-related genes, which dramatically decreased on PLCL-5SC after culturing for 120 hours (Figure 15(d)). However, other differentiation-related genes,* COL2A1* and* PPAR*_*γ*_ *2*, could not be detected under any of the culture conditions.

## 4. Discussion

The novel PLCL-SC electrospun membranes exhibited biodegradable and biocompatible properties, which could support the attachment, proliferation, and differentiation of the hWJMSC. Although various electrospun membranes have been previously reported to react as specific matrices for neurons and promoted neurite outgrowth* in vitro* [21], none has been tested for hWJMSC differentiation. This study revealed for the first time that the SC content of the copolymer membrane influenced neuronal differentiation of hWJMSC by membrane surface characteristics, which increased biocompatibility. SC content in the PLCL blend was apparently the key adjustable factor to control the membrane's rate of biodegradability. This enables the membrane's persistency to be controlled, when providing a substrate for tissue formation and renewal.

### 4.1. Characteristic of PLCL-SC Membranes

The surface texture of the matrices played important roles affecting attachment, proliferation, and the spreading ability of the targeted cells. Fiber diameter also primarily related to the porosity of the membranes (small diameter, less porosity) [37]. The smallest fiber diameter on PLCL-2.5SC may arise from increased membrane porosity (up to 48–53%) due to the water-soluble nature of the SC protein [38]. In addition, the SC resulted in more random alignment of the fibers compared with PLCL alone which had greater merging of fibers. The new amide groups (C=O, N–H, and C–N) detected on the surface of PLCL-SC but not on the PLCL were reported to enhance biocompatibility [39]. Surfaces with positively charged amines promote the exposure of high-density bound receptors, as well as focal adhesion components, by absorption of fibronectin [40]. The activation of these surface functional groups has been demonstrated in several human cell types, for example, endothelial cell growth, osteoblast differentiation and mineralization [41], and fibroblast adhesion, proliferation, and matrix formation [42]. The membrane surface and also SC blending apparently affect the hydration side chain of PLCL in its native state [43], deduced from increased *T*_*m*_ and *T*_*c*_. Higher *T*_*m*_ relates to higher membrane quality and greater thermodynamic stability of proteins [44], which would be very helpful in determining the stability of a potential implant at body temperature [45]. Whereas *T*_*c*_ data is necessary in pharmaceutical polymer industry to indicate well-characterized drug delivery compounds, amorphous membranes are usually desirable to process drugs at temperatures below the *T*_*c*_ point [46].

Hydrophilicity is a major determinant of degradation rate of biomaterials; the higher it is, the faster the materials degrade [47, 48]. The effects of SC on hydrophilicity of the copolymer membrane were verified by the swelling properties and water contact angle, which were higher for PLCL-SC than for PLCL. Normally, the degradation rate of poly(*ε*-caprolactone) (PCL) is very slow. It takes more than a year for complete degradation to occur [49]. Higher concentrations of SC accelerated degradation, SC release, and weight loss. The degradation of this copolymer was assumed to be a random chain scission. The adsorption of water molecules on the polymer surface was probably the first step in hydrolytic process, followed by ester hydrolysis of the polymer matrices [50]. Swelling of PLCL in water may have been due to water adsorption or entrapment of air bubbles between the fibers [51].

Due to the high hydrophilicity of SC, the membrane fibers continuously released SC. During degradation, hydroxyl groups were released from the hydrophilic side chains of the SC molecules, whilst carboxylic acid was produced from physiological degradation via hydrolysis of PLCL [52]. Although the isoelectric point of SC (around 4) was lower than that of the buffer, the pH of the buffer changed only slightly. This may have been due to an equilibrium interaction between the OH^−^ (negative charge) and the H+ (positive charge), released from the SC component into the buffer [53].

### 4.2. Cytotoxicity, Cellular Activities, and Differentiation of WJMSC on PLCL-SC Membranes

Some studies reported that SC enhances mammalian cell proliferation [54] and adhesion [38] and is involved in breast cancer cell migration [55]. This study provided the first evidence that SC improves proliferation efficiency of WJMSC, at the nontoxic concentration below 0.25% (w/v), agreeing with previous studies of other cell types [1].

Initial cell attachment plays an important role in further cell viability on biomaterials; thus the pattern of architecture and surface properties of the materials are crucial [29]. Although most of the observed WJMSC of this study appeared as separated, single, attached cells, some colonies were still found on the surfaces. Colony formation may influence the expression of several groups of genes, following modification of cellular morphology and behavior. Clumps of MSC usually successively undergo cellular differentiation [56]. Enhanced cell attachment and proliferation on PLCL-SC membranes were verified by the upregulation of protein markers, cyclin A and FAK. Application and study of SC in cell culture were previously reported on a few cell types. Proliferation of human skin fibroblasts was increased 250% on PS culture vessels precoated by SC [57]. The 2D- and 3D-scaffold of SC-gelatin activated feline fibroblast proliferation and attachment on days 1 and 4 of cultures, without arresting the cell cycle [58]. Nevertheless, how the biochemical composition of SC enhances cell attachment and proliferation is unclear. Possibly SC contains anionic amino acids, such as glutamic and aspartic acid, which might provide a favorable surface for cell attachment [57]. Physiological interactions between the cells and the surface properties of the copolymer membranes may affect cell attachment and proliferation. Cells preferred to adhere and proliferate on PLCL-SC, more than on PLCL, because of thinner and finer fiber characteristics [59]. Moreover, PLCL-SC membranes supplied a large surface area and multiple focal adhesion points, by being more hydrophilic and porous, which indirectly increased mechanotransductive signaling (attachment and cytoskeleton-related), such as integrin-related signaling, extracellular receptor kinase, and FAK [60].

Stem cell fate and differentiation was influenced by the cell-matrix interaction, similar to what happened on the extracellular matrix, by the nanoscale features [61], topography, and microstructure [60]. The novel PLCL-SC membranes of this study exhibited the potential to control or induce hWJMSC differentiation into neuronal lineage by upregulating expression of* nestin*. Neural differentiation in this study was presumably due to the mechanical induction of fiber size and alignment, similar to that reported for hESCs [62]. Many kinds of electrospun scaffold directly stimulate stem cell and progenitor cell differentiation to neurons and promote and guide neurite outgrowth [63–65]. However, PLCL has never been reported to induce cellular differentiation, since it was first developed for vascular and skin tissue engineering [66]. Increased neuronal differentiation specificity in the PLCL-SC membrane might be related to different kinds of amino acids contained in SC in varying proportions [38]. A higher density of amino acids on the surface of culture vessels enhanced neuronal differentiation and attachment [67]. However, culture time of the cells on membranes in this experiment may not have been long enough to activate the mechanisms of* COL2A1* (chondrogenic differentiation) [68] and* PPAR*_*γ*_ *2* (adipogenic differentiation) [69] expression. Even though the cells expressed* nestin* and decreased stemness gene expressions, the expression patterns were still unclear. This novel copolymer maintained hWJMSC stemness and also initiated neuronal differentiation. The membrane did not stimulate chondrocytes or adipocytes differentiation, at least in the experiment's initial stages. These effects were assumed to be due to interactions between the material properties and hWJMSC, which will be useful for the advancement of tissue engineering for neuronal related applications.

## 5. Conclusion

This study reported for the first time that SC promoted hWJMSC proliferation at noncytotoxic concentrations. Also, new biodegradable PLCL-SC copolymer membranes were successfully fabricated by electrospinning. The improved membranes, which exhibited better qualities, resulted in enhancing hWJMSC adhesion and proliferation. Differentiation towards neuronal lineage was initiated by the membrane topography. Therefore, this study revealed that PLCL-SC membranes are useful additions to medical stem cell and tissue engineering technologies.

## Figures and Tables

**Table 1:** The primers sequences.

Gene	Accession number	Sequence (5′ → 3′)
*POU5F1*	NM_002701	Fw CCATTTTGGTACCCCAGGCT
		Rv ACTGTGTCCCAGGCTTCTTT

*SSEA-4*	NM_006927	Fw CCCAGATGGTATTGGACACA
		Rv GCCCTAGAACTCCAATCACA

*Nestin*	NM_006617	Fw GGAAGAGGTGATGGAACCAC
		Rv TCCTGCTCGCTCTCTACTTT

*COL2A1*	NM_001844	Fw GTCCTCTGCGACGACATAAT
		Rv CCCATTGGTCCTTGCATTAC

*PPAR*_*γ*_ *2*	NM_138712	Fw CCTATTGACCCAGAAAGCGATTC
		Rv GCATTATGAGACATCCCCACTGC

*GAPDH*	NM_002046	Fw TGCTGGCGCTGAGTACGCG
		Rv TGACCTTGGCCAGGGGTGCT

**Table 2:** Fiber diameter and percentage of porosity of the five types of PLCL-SC membranes at various concentration of SC. Statistical difference is indicated by lowercase letters (a, b, c, d and e) (*p* ≤ 0.05).

SC concentration in membranes (% w/v)	Fiber diameter (nm)	% porosity
0	501 ± 24^b^	69.5 ± 0.8^d^
2.5	421 ± 12^a^	48.7 ± 0.5^a^
5.0	418 ± 18^a^	53.1 ± 0.7^b^
7.5	438 ± 16^a^	59.4 ± 0.7^c^
10.0	456 ± 24^ab^	73.1 ± 1.5^e^

**Table 3:** FT-IR spectral and analyzed functional groups from the membranes.

Peaks (cm^−1^)				Functional groups
PCL membrane	Sericin powder	PCL-2.5SC membrane	PCL-5SC membrane	
—	3283	3291	3284	N–H stretching
2942	—	2944	2944	C–H stretching
1758	—	1761	1759	C=O stretching
—	1648	1657	1655	C=O stretching (amide I)
—	1522	1534	1534	N–H bending (amide II)
1453	—	1455	1455	CH_2_ bending
1368	1397	1368	1368	CH_3_ bending
—	1241	1240	1240	C–N stretching (amide III)
1185	—	1183	1184	C–O stretching

**Table 4:** DSC thermogram from the membranes.

Membranes	*T*_*m*_ (°C)	*T*_*c*_ (°C)
PLCL	157	98.3
PLCL-2.5SC	160.1	108.4
PLCL-5SC	161.4	102.1
